# What is needed to guide testing for anorectal and pharyngeal *Chlamydia trachomatis* and *Neisseria gonorrhoeae* in women and men? Evidence and opinion

**DOI:** 10.1186/s12879-015-1280-6

**Published:** 2015-11-17

**Authors:** Nicole H. T. M. Dukers-Muijrers, Julius Schachter, Genevieve A. F. S. van Liere, Petra F. G. Wolffs, Christian J. P. A. Hoebe

**Affiliations:** Department of Sexual Health, Infectious Diseases and Environmental Health, South Limburg Public Health Service, Geleenbeeklaan 2, 6166 GR Sittard-Geleen, The Netherlands; Department of Medical Microbiology, School of Public Health and Primary Care (CAPHRI), Maastricht University Medical Centre (MUMC+), Maastricht, The Netherlands; Department of Laboratory Medicine, University of California, San Francisco, CA USA

**Keywords:** Extra-genital, *Chlamydia trachomatis*, *Neisseria gonorrhoeae*, Women, MSM

## Abstract

**Background:**

Anorectal and pharyngeal infections with *Chlamydia trachomatis* (CT) and *Neisseria gonorrheae* (NG) are commonly observed in men who have sex with men (MSM). There is increasing evidence that such infections at extra-genital sites are also common in women. In both sexes, these infections are largely overlooked as they are not routinely tested for in regular care. Testing based on sexual behavior or symptoms would only detect half of these extra-genital infections. This paper elucidates the differences and similarities between women and MSM, regarding the epidemiology of extra-genital CT and NG. It discusses the clinical and public health impact of untested extra-genital infections, how this may impact management strategies, and thereby identifies key research areas.

**Discussion:**

Extra-genital CT is as common in women as it is in MSM; NG in women is as common at their extra-genital sites as it is at their genital sites. The substantial numbers of extra-genital CT and NG being missed in women and MSM indicate a need to test and treat more patients and perhaps different choices in treatment and partner management strategies. Doing so will likely contribute to reduced morbidity and transmission in both sexes. However, in our opinion, it is clear that there are several knowledge gaps in understanding the clinical and public health impact of extra-genital CT and NG. Key research areas that need to be addressed concern associated morbidity (anorectal and reproductive morbidity due to extra-genital infections), ‘the best’ management strategies, including testing and treatment for extra-genital CT, extra-genital treatment resistance, transmission probabilities between partners and between anatomic sites in a woman, and impact on transmission of other infections. Data are also lacking on cost-effectiveness of pharyngeal testing, and of NG testing and anorectal CT testing in women. Gaps in the management of extra-genital CT and NG may also apply for other STIs, such *Mycoplasma genitalium*.

**Summary:**

Current management strategies, including testing, to address extra-genital CT and NG in both sexes are suboptimal. Comparative data on several identified key themes in women and MSM are lacking and urgently needed to guide better management of extra-genital infections.

## Background

It is a continuous challenge to control the spread of sexually transmitted infections (STIs). *Chlamydia trachomatis* (CT) and *Neisseria gonorrhoeae* (NG) are sexually transmissible bacteria that may result in serious complications such as pelvic inflammatory disease, infertility and ectopic pregnancy in women, and epididymitis in men and play an important role in enhancing HIV transmission [1, 2]. The Centers for Disease Control and Prevention (CDC) reported about 1.4 million new CT infections in 2013 and numbers are still increasing each year [3]. In Europe, the number of CT infections is increasing with more than 250 000 new cases reported each year [4].

The use of highly sensitive and specific laboratory assays, i.e. nucleic acid amplification tests (NAAT), has revealed the frequent presence of CT and NG at extra-genital sites. Infections at extra-genital sites are common in both men who have sex with men (MSM) and women. In MSM, this led to specific control guidelines, including expanded testing [5–9]. In women, such guidelines are beginning to emerge. However, the occurrence of extra-genital infections also has led to international debate on the (need for) control of such infections by testing and treatment.

This paper addresses the state of the art in extra-genital NG and CT epidemiology, elucidating the differences and similarities in women versus MSM, argues how this may impact control strategies, and identifies the knowledge gaps to address these issues to guide testing.

### Epidemiology of extra-genital STI in women and MSM

To provide an overview on state-of-the-art on the prevalence and anatomic site distribution of CT and NG infections in women, we have reviewed the literature focusing on including studies (in English) in women describing anorectal and/or pharyngeal CT and NG infections detected using NAATs. We conducted a Medline search (last update 8 June 2015) using the terms: (‘women or woman or female’) and (‘chlamydia’, ‘gonorrhea’ or ‘gonorrhoeae’). These terms were used in combination with (‘anorectal or rectal or anal’) or (‘oropharyngeal or pharyngeal or oral’) or (‘extra-genital’). Additionally, relevant results on anorectal CT that could not directly be retrieved from these papers were kindly provided by the authors of these studies. We compared results to findings in several key papers in MSM from different countries and using NAAT. Because there is an abundant literature on extra-genital infections in MSM a literature search was deemed not necessary.

#### Prevalence

Prevalence, which is defined here as the proportion of the study population that tested positive, has so far for women only been assessed in clinic-based populations. The observed prevalence ranges of extra-genital CT as detected by NAAT are notably similar between women [10–32] and MSM [11, 18, 24, 25, 32–39], ranging between 1 and 3 % for pharyngeal CT and 1–18 % for anorectal CT (see Table 1). Of note, MSM who define themselves as being heterosexual (e.g. male swingers) have appreciable numbers of anorectal infections as well [11]. Pharyngeal CT and NG prevalences in heterosexual men are similar to that seen in women (e.g. [11, 28]). Overall however, data in heterosexual men are scarce.

**Table 1:** Prevalence of extra-genital *Chlamydia trachomatis* (CT) and *Neisseria gonorrhoeae* (NG) in women and in men who have sex with men (ref.: [10–32] for women and [11, 18, 24, 25, 32–41] for MSM)

	Women		Men who have sex with men		
	CT	NG	CT	LGV (of CT+)	NG
Pharyngeal	1–3 %	1–2 %	1–3 %	9–16 %	4–12 %
Anorectal	7–17 %	0–3 %	1–18 %	2–16 %	6–21 %
Genital	5–13 %	1–2 %	3–8 %	2 %	3–11 %

*LGV Lymphomgranuloma Venereum*

Most studies on anorectal CT (see Table 2) have included women tested on indication of receptive anal intercourse (RAI). Few studies reported systematic testing of all women, i.e. testing irrespective of reported behavior or symptoms [10–12, 27]. Most studies were done in women attending care services; one study reported on women testing by self-triage via the Internet [14] and another study reported on women in the ‘open population’ reached via friends in their social network [31]. Comparable prevalence ranges in the tested populations were reported across studies. It was notable that some geographic variation might be present. Excluding two studies that tested on indication of genital CT, prevalences of anorectal CT in most studies in Canada (11.7–13.5 % ) or US (5.1–27.3 % ) appeared even higher than that of most studies in Europe (5.6–12.5 % ). Still, we need more representative data from future studies with less selected populations to be able to compare prevalence rates (and associated factors) in women. Such population-based studies are feasible given the high acceptance of self-collection methods for testing.

**Table 2:** Overview of studies that include anorectal *Chlamydia trachomatis* (CT) nucleic acid amplification testing in women by routine systematic testing or selective testing on indication of receptive anal sex (RAI) or otherwise

	Setting	Population	Tested *N*	Anorectal CT % (*n*)	Had RAI % (*n*)	Not had RAI % (*n*)	Anorectal CT In women with RAI % (*n*)	Anorectal CT In women without RAI % (*n*)	Genital CT % (*n*)	Genital and/or anorectal CT	Single anorectal CT in genital and/or anorectal positives	Single anorectal CT in anorectal positives
	*Routine systematic testing*											
van Liere et al. [10]	STI clinic, South Limburg, Netherlands ’12–‘13	All	654	8.4 % (55/654)	31.0 % @6 (203/654)	69.0 % @6 (451/654)	7.9 % (16/203)	8.6 % (39/451)	11.2 % (73/654)	11.6 % (76/654)	3.9 % (3/76)	5.4 % (3/55)
Van Liere et al. [11]	STI clinic, South Limburg, Netherlands ’10–‘12	Swingers	461	6.7 % (31/461)	29.5 % @6 (136/461)	70.5 % @6 (325/461)	3.5 % (16/136)	4.6 % (15/325)	6.3 % (29/461)	7.8 % (36/461)	19.4 % (7/36)	22.6 % (7/31)
Peters et al. [12]	Primary health care facilities South Africa ’11–‘12	All	603	7.1 % (43/603)	4.3 % @6 (26/603)	95.7 % @6 (577/603)	3.8 % (1/26)	7.3 % (42/577)	16.0 % (96/603)	17.7 % (107/603)	10.3 % (11/107)	25.6 % (11/43)
Ostergaard et al. [27]	STI clinic, Denmark ’95–‘96	All	196	5.6 % (11/196)	43.9 % @e (86/196)	56.1 % @e (110/196)	4.7 % (4/86)	6.4 % (7/110)	14.5 % (25/173)	15.6 % (27/173)	7.4 % (2/27)	18.2 % (2/11)
	*Testing on indication of RAI*											
Trebach et al. [28]	2 public health STI clinics, Baltimore, USA ’11–‘13	Had RAI, sharing toys	602	8.6 % (52/602)	100 % @3	0%	8.6 % (52/602)	N/A	9.4 % (50/532)	11.8 % (63/532)	25.4 % (13/63)	26.0 % (13/50)
Bachmann et al. [24]	STI clinics, hospital-based HIV clinics, USA ’03–‘07	Had RAI, STD contact	99	27.3 % (27/99)	40.4 % @2 (40/99)	59.6 % @2 (59/99)	17.5b % (7/40)	33.9 % (20/59)	23.2 % (23/99)	30.3 % (30/99)	23.3 % (7/30)	25.9 % (7/27)
Van der Helm et al. [25]	STI clinics, Amsterdam, South Limburg, Netherlands, ’06–‘07	Had RAI	901	9.3 % (84/901)	100 % @6	0 %	9.3 % (84/901)	N/A	N/A	N/A	N/A	N/A
Sethupathi et al. [19]	STI clinic Singleton hospital, UK ’06–‘08	Had RAI, STD contact, symptoms, assault	160	12.5 % (20/160)	51.2 % @u (82/160)	48.8 % @u (78/160)	12.2 % (10/82)	12.8 % (10/78)	14.1 % (22/156)	14.7 % (23/156)	4.3 % (1/23)	5.0 % (1/20)
Koedijk et al. [18]	STI clinics Netherlands ’06–‘10	Had RAI, symptoms, prostitution	18,238	9.3 % (1695/18,238)	N/A	N/A	N/A	N/A	9.4 % (1709/18,238)	11.7 % (2139/18,238)	20.1 % (430/2139)	25.4 % (430/1695)
Hunte et al. [16]	STI clinic Miami USA ‘07	Had RAI	97	17.5 % (17/97)	100 % @3	0 %	17.5 % (17/97)	N/A	16.5 % (16/97)	17.5 % (17/97)	5.9 % (1/17)	5.9 % (1/17)
Peters et al. [22]	STI clinic, The Hague, Netherlands,’07–‘08	Had RAI	850	8.8 % (75/850)	100 % @6	0 %	8.8 % (75/850)	N/A	8.9 % (76/850)	10.8 % (92/850)	20.7 % (16/92)	21.3 % (16/75)
Javanbakt et al. [17]	STI clinics USA ’08–‘10	Had RAI	1203	14.6 % (171/1203)	100 % @3	0 %	14.6 % (171/1203)	N/A	12.0 % (144/1203)	16.0 % (193/1203)	25.4 % (49/193)	28.7 % (49/171)
Shaw et al. [23]	STI clinic UK, before ‘13	Had RAI	312	7.1 % (22/312)	100 % @u	0 %	7.1 % (22/312)	N/A	6.7 % (194/3043)	N/A	N/A	22.7 % (5/22)
Cosentino et al. [29]	STI clinic Health department; HIV clinic, Pittsburgh, USA ’09–‘10	Had RAI	272	7.7 % (21/272)	100 % @e	0 %	7.7 % (21/272)	N/A	N/A	N/A	N/A	N/A
Garner et al. [15]	Manchester Centre for Sexual Health, UK ‘10	Had RAI	91	6.6 % (6/91)	100 % @u	0 %	6.6 % (6/91)	N/A	N/A	9.4 % (59/631)	N/A	16.7 % (1/6)
Bazan et al. [13]	Student health clinic Seattle, before ‘93	Had RAI	341	13.5 % (46/341)	100 % @12	0 %	13.5 % (46/341)	N/A	N/A	14.7 % (49/334)	12.2 % (6/49)	13.6 % (6/44)
	*Testing on indication of genital CT*											
Ding et al. [26]	STI clinic Plymouth, UK ’12–‘13	Had genital CT	97	77.3 % (75/97)	25.8 % @u (25/97)	74.2 % @u (72/97)	80.0 % (20/25)	76.4 % (55/72)	100 %	100 %	N/A	N/A
Musil et al. [30]	Canberra Sexual Health Centre, Australia ’13–’14	Had genital CT, contact, symptoms	56	57.1 % (32/56)	33.9 % @6 (19/56)	66.1 % @6 (37/56)	57.9 % (11/19)	56.8 % (21/37)	76.8 % (43/56)	78.6 % (44/56)	2.3 % (1/44)	3.1 % (1/32)
	*Testing on indication of Pelvic examination (PE)*											
Gratrix et al. [21]	STI clinic, Calgary, Canada ‘12	Received PE	1570	11.7 % (183/1570)	N/A	N/A	N/A	N/A	7.1 % (110/1543)	N/A	N/A	N/A
Gratrix et al. [21]	STI clinic, Edmonton, Canada. ‘12	Received PE	1485	13.5 % (201/1485)	N/A	N/A	N/A	N/A	12.6 % (177/1403)	N/A	N/A	N/A
Barry et al. [20]	STI clinic, San Francisco, USA, 07–‘08	Received PE	1308	5.1 % (67/1308)	21.8 % @3 (256/1173)	78.2 % @3 (917/1173)	4.3 % (11/256)	4.8 % (44/917)	5.9 % (76/1308)	6.7 % (88/1308)	15.9 % (14/88)	21.8 % (14/67)
	*Self-triage Internet*											
Ladd et al. [14]	Internet iwantthekit.org, USA ’09–‘11	Self-request	205	12.7 % (26/205)	57.5 % @3 (118/205)	42.5 % @3 (87/205)	N/A	N/A	17.6 % (35/201)	N/A	N/A	N/A
	*Peer-intervention (social network)*											
Dukers-Muijrers et al. [31]	‘Open population’ South Limburg Netherlands, ’13–14	Test provided by a friend (social network)	58	6.9 % (4/58)	N/A	N/A	N/A	N/A	6.9 % (4/58)	8.6 % (5/58)	20.0 % (1/5)	25.0 % (1/4)

*N/A* Not Available, *RAI* receptive anal intercourse @ reported in the past 2, 3, 6, or 12 months;@ u: reporting period unknown @ e: reported ever, *PE* pelvic examination

In MSM in the Netherlands and Germany, lymphogranuloma venereum (LGV) serovars comprised 2–16 % of CT positive anorectal samples and were also found in the pharynx and urethra [40, 41]. In women, LGV is very unusual, although systematic assessment of LGV in women is scarce [42].

The prevalence of extra-genital NG reflects the well known disparity between MSM and women (see Table 1). The occurrence of NG is at all anatomic sites substantially less frequent in women; women show NG prevalences up to 3 %, while prevalences in MSM are up to 21 % [10–18, 20–25, 28, 29, 33, 34, 36–39].

#### Concurrence of infections at extra-genital and genital sites

There are notable differences between women and MSM in how frequent an infection at the extra-genital site occurs together with infection at the genital site. A women with an anorectal infection usually also has a concurrent genital infection. The studies presented in Table 2 show that in women, between 5 and 29 % of the anorectal CT infections are single site anorectal infections, i.e. without a genital infection.

Among the women who have a CT or NG infection at the genital site (which is routinely tested for in care), a substantial part have a concurrent infection at the anorectal site: between 33 % (i.e. [12]) and 83 % (i.e. [11]) of women with genital CT also were found to have an anorectal CT (calculated from the studies presented in Table 2).

In contrast, MSM with anorectal infections usually do not have a concurrent infection at the genital site. In MSM anorectal infections are mostly single site infections, i.e. up to 91 % for CT and up to 70 % for NG [33–39, 43]. Still, similar to women who have a genital infection, a large proportion of MSM who have a genital infection may have a concurrent infection at the anorectal site: one study of 2436 MSM in a Dutch STI clinic found anorectal infections in about half of genitally infected MSM [43].

Pharyngeal CT prevalence is low in MSM and in women (see Table 1), and in both sexes frequently occurs in the absence of anorectal or genital CT, e.g. of pharyngeal CT 32–44 % was single in women and 53–85 % in MSM [18, 32, 33, 43–47]. Pharyngeal NG prevalence is higher in MSM than in women (see Table 1), and also frequently occurs as a single infection in both sexes. For example one study revealed 53 % of pharyngeal NG infections were single in MSM and 73 % in women [43].

### Current management

#### Testing practices

Detection of extra-genital CT and NG is best done by NAAT [7]. Such tests are highly sensitive and specific and were shown valid and robust for extra-genital detection (e.g. [25, 29, 48, 49]). Also, self-collection of samples in case of anorectal infections is well accepted and feasible in both women and in men. Still, the lack of clearance from the Food and Drug Administration (FDA-USA regulations) and lack of a CE-IVD mark (European regulations) for such testing, has greatly hampered its use in current clinical practice [50].

The focus in current health care is *not* on extra-genital testing; it has largely remained on genital testing. For example, a laboratory surveillance from all types of health care providers who perform CT testing in Australia showed that only in 3 % of the test episodes was extra-genital CT tested alongside genital CT [51]. This is further corroborated by a study from The Netherlands showing that GPs and gynecologists performed more CT tests than did STI clinics, but they infrequently (<1 % ) tested for anorectal or pharyngeal CT [52]. Extra-genital testing, when it is done, is done in the STI clinic setting, and CT and NG are usually tested simultaneously because most commercially available NAATs detect both. However, such extra-genital testing tends to focus on MSM, not women, and test practices vary widely between STI clinics, even within a country [18, 34, 53].

#### Testing guidelines

Current international guidelines for CT and NG testing [5–9] include a pharyngeal or anorectal test after symptoms and after behavioral exposure, i.e. receptive oral sex or receptive anal sex, respectively. Most guidelines focus on MSM and some also include specific groups of women, such as sex workers. None of the guidelines include women in general as a target group. Unfortunately, the recommendation to restrict testing to certain exposure risks has not been based on evidence. Actual testing results show that extra-genital testing of all people reporting extra-genital exposure would still result in over half of extra-genital infections remaining undetected in both women (see Table 2) and in MSM who attend health care for genital testing [11, 18, 24, 25, 32–39, 43–47]. The prevalence of anorectal CT was consistently similar between women reporting and not reporting (receptive) anal sex, as shown in Table 2. Similar observations have been made for NG, and also in MSM. Could the observed presence of anorectal infections be explained by underreporting of anal sexual exposure? Reporting bias is unlikely to explain a major part as then such bias would need to be unrealistically high and consistent across studies and countries. Bias by using a recall period that is too short to capture the behavioral exposure is probably more likely, given that anal sex is usually recorded as behavior in a given time period (usually a couple of months). Oral and anal sex are commonly reported by women and MSM [10, 11, 20, 26, 27, 54]. Therefore, infections may have been acquired before the beginning of the screening interval, and be unnoticed due to its asymptomatic nature and lack of extra-genital testing. It has been suggested that anorectal pathogens may potentially be transmitted by practices that involve contact with the anus other than penetrative anal-genital sex (i.e. by transmission by fingers or by sex toys), although evidence for this is inconclusive [10, 30, 55].

It should be noted that for anorectal infections, transmission methods other than sex with by an infected partner have been postulated. The most plausible of these in women is self-infection (auto-inoculation). The presence of anorectal infections in women without anal sex and the high rate of concurrence with infections at the genital site, fuelled the belief that anorectal infections in women are largely the result of self-infection. The anatomical proximity of genital and anorectal site makes this plausible. However, self-infection has not been confirmed by rigorous data. Also, the observed high rates of concurring genital and anorectal infections in women is predictable given the likelihood that women who have anal sex will also have vaginal sex [54]. Possibly differences in behavior between the women and MSM (frequency of anal sex, number of anal sex partners, co-practice of anal and genital intercourse) may also drive discordance of anatomic site infections. Further, it can be considered very unlikely that possible swab contamination by inadequate swab handling (due to contamination from the genital infection or the environment), would contribute much to the observed anorectal detections [25, 56]. A different theory to explain anorectal CT detection involves the gastro-intestinal (GI) tract as a reservoir for CT detection. While asymptomatic CT infections have been detected in the GI tract of neonates exposed at birth, and rectal shedding has been observed in children in trachoma endemic areas, the theory that the GI tract could act as a reservoir in humans, for example though oral exposure, was more recently shaped by new observations in mice [57–59]. However, evidence to support long term persistence of CT in the GI tract in human adults is completely lacking.

The vast majority of extra-genital CT and NG are asymptomatic, i.e. without oral or anorectal symptoms reported [1, 2]. Even in the case for LGV, many infections in MSM have been shown to go without symptoms [40, 41]. In fact, this is similar to genital infections, that are also frequently asymptomatic. There is little or no evidence that CT strains differ between anatomic sites regarding their genotype or their pathogenic potential. In MSM, some studies demonstrated associations between anorectal NG infections, anorectal NG bacterial load and proctitis [35, 47, 60]. Still, it should be noted that generally anorectal symptoms are rarely reported (<5 %) and that most extra-genital infections are without symptoms.

#### Treatment

Generally, treatments recommended for extra-genital STI do not differ from that of genital STI. For CT however, the recommended first choice treatments may differ between anorectal and genital infection. For genital CT infection azithromycin 1-g single dose is advised. For anorectal CT, azithromycin or doxycycline 100mg twice daily for seven days are considered equal first line treatments in the US. Other countries (Netherlands, Australia, and UK in their most recent draft of guidelines) now have doxycycline as first choice in anorectal CT [5–9]. These new recommendations reflect concern that azithromycin efficacy for anorectal infections may be less than what was expected. Yet, neither recommendation, i.e. to use the treatments as equal first line treatments or prefer the one over the other, stems from robust studies showing equivalent or different efficacy at different anatomic sites. These recommendations are extrapolated from solid evidence supporting the efficacy of these regimens for genital CT treatment and from clinical experience and expert consultation [61–63]. The internationally recommended treatment regimen for LGV is a 3-week course of oral doxycycline 100 mg twice daily [55]. Genital and extra-genital NG can be treated with ceftriaxone or with ciprofloxacin when ceftriaxone is contraindicated and strains show no ciprofloxacin resistance [5–9].

## Discussion

Routine extra-genital testing for CT and NG is possible using NAAT and minimally invasive sampling methods. However, extra-genital infections are still not routinely tested for in MSM and testing is even less common in women. As these infections are mostly asymptomatic, they are frequently overlooked in health care. Extra-genital infections, in women especially anorectal CT, are common and testing based on exposure or symptoms misses over half of these extra-genital infections. More testing in women and in MSM would identify many more extra-genital infections. But, what are the implications for clinical practice of testing more? Untreated infections might continue to cause complications and potentially drive further transmission. Still, what are the public health gains of testing more? Should control strategies be different for MSM and women? Some answers to these questions will provide us with more insight into what is needed to guide extra-genital testing in MSM and in women.

### What are implications for clinical practice of managing extra-genital CT and NG?

#### Testing more patients and testing more samples per patient

A substantial proportion of infected individuals do not attend a testing facility for testing or retesting, and are obviously missed, hence remain untreated [64]. Even so, when people do attend care, they are in most cases tested at genital sites only. Addressing currently hidden CT and NG by extra-genital testing of more persons would reveal many more extra-genital infections. Several approaches to increase testing coverage for genital and anorectal infections have been evaluated and found feasible. These include using self-collected samples at home, and using automated e-health programs [14, 31, 65, 66].

Of patients who have an anorectal infection, women frequently have a concurrent infection at the genital site while MSM frequently do not have a concurrent genital infection. This implies that adding a extra-genital test to routine genital testing would reveal much more additional infected individual MSM than it would for women, even when the prevalence is similar (such as in anorectal CT). This also implies that to detect large part of extra-genital infections in women we could just test women with genital infections. A strategy to achieve this, without having to ask the women to come back for extra-genital testing, could be to directly take a extra-genital sample at the routine genital STI testing visit, but to only test the extra-genital specimen from women who tested positive for their genital infection. In such case, fewer extra-genital laboratory tests would have to be performed compared to routine testing of both specimens in women, perhaps reducing costs. Yet, such phased testing strategy would also demand more complex laboratory logistics and increase time to return test results. Using this strategy implies there is a reason to detect the extra-genital infection in a women who will be treated because of her genital infection (different treatment regimen or different partner management needed?). Also, such phased testing strategy would not detect the single site extra-genital infections that still will remain undiagnosed and untreated. Another testing strategy potentially reducing costs is to test pooled multiple site samples. Such approach precludes feedback on the anatomic site(s) where the infection(s) occurs precluding treatment guidance, and its effectiveness needs to be further explored.

More information on the clinical and public health impact is needed to decide what would be the optimal cost-effective algorithm for testing. This clinical and public health impact of leaving extra-genital infections untested may be different between the two sexes. This is because the co-occurrence of infections at multiple anatomic sites differs between MSM and women and may impact the need for additional management strategies. The clinical and public health impact also is dependent on the (yet unknown) role of extra-genital infections in morbidity and transmission.

Further insight on these issues is essential to know how the additional costs of testing extra-genital samples and managing more identified positive cases balances the costs avoided by reducing spread and morbidity due to extra-genital testing.

#### Making different treatment choices

If treatment for genital infections is also adequate for extra-genital infections, then part of the extra-genital CT and NG would be ‘inadvertently treated’ by treating the infection at the genital site (that is usually routinely screened). This would most frequently apply to anorectal infections in women, as these usually co-occur with a genital infection, reducing the negative consequences (in terms of morbidity and transmission) of leaving anorectal CT and NG infections in women untested. However, this scenario may not be true for anorectal CT. There are reports suggesting that doxycycline is a better treatment for anorectal CT than is azithromycin [61–63]. If a clinician knew the patient also had an anorectal CT, a different treatment choice might be made. At the moment, we lack thorough studies to decide what is the best treatment for anorectal CT. Restrictions in financial means for extra-genital testing has led some clinicians to taking a pragmatic approach by directly treating genital CT positive patients with doxycycline, assuming this will treat a potential concurrent anorectal infection better than when using azithromycin. Still, the strategy to use doxycycline in anorectal CT is not based on solid evidence. Such strategy is also limited by the higher non-compliance rates seen with doxycycline use because of its longer treatment duration [67]. Until we better understand the effectiveness of anorectal CT treatment regimens, the impact of not testing for anorectal CT in women is difficult to assess. Well designed randomized controlled trials on this topic are urgently needed.

In MSM, an additional contributing factor to increasing the adverse consequences of an untested extra-genital CT, is the occurrence of LGV. LGV prevalence is low, and there are marked differences in geographic distribution, but it requires additional and different testing and treatment.

#### Managing so far undetected treatment resistance

The introduction of NG treatment resistant strains poses a challenge for its management [68]. So far, there are no reports that its occurrence is different for genital or extra-genital NG, although it has been suggested that pharyngeal NG may act as a reservoir for resistance [69]. Not (appropriately) treating extra-genital NG may increase the spread of resistant NG in the population, especially in MSM who show a higher prevalence than women. Mathematical modeling suggested that the most effective control strategy for treatment resistant NG is by following up treated infections to re-treat failures, rather than just testing and treating more patients [70]. This could equally apply for extra-genital infections.

For CT, there is yet no evidence of homotypic anti-microbial resistance [71]. Still, our understanding is limited as testing for antimicrobial resistance for CT is not routinely available, although whole genome sequencing provides new opportunities [72].

#### Making more customized patient and partner management choices

Management strategies such as re-testing, partner notification and expedited or accelerated partner treatment have been shown to be highly effective strategies to prevent relatively high numbers of CT and NG [73]. Partners of positive patients and previously tested positive patients have a greater risk to also test positive. Treatment of the sexual partners of positive cases is therefore recommended (partner-notification) and infected patients themselves are advised to be re-tested between 3–12 months post-treatment to detect a new CT or NG infection [3–9]. While for CT, the usefulness of performing a test-of-cure can be seriously questioned [74, 75], for NG, a test-of-cure may sometimes be recommended to detect a persistent infection [3–9] and as recently suggested, to manage treatment resistant NG [70]. Again, extra-genital infections may in part be incidentally handled by the strategies deployed for a detected genital infection. However, it is not clear whether such incidental handling is effective for extra-genital infections. For example, partner notification may not be applied when the patient did have unprotected anal/oral sex but unprotected genital sex was not reported and a test-of-cure may be negative for a genital NG, while it may be positive for (untested) anorectal or pharyngeal NG.

It also is apparent that by not testing, a single extra-genital infection would always be missed and none of the treatment, retest or partner notification strategies would be employed. Therefore, by testing and treating at genital sites only, essential control opportunities in the management of both female and MSM patients and their partners are lost.

### What are public health gains by managing extra-genital CT and NG?

#### Avoided morbidity

Some studies in MSM linked anorectal NG to symptomatic proctitis, which is an inflammatory syndrome of the distal 10–12 cm of the rectum. Some LGV strains cause a severe proctocolitis [55]. In women, it is yet unknown whether anorectal infections can cause anal symptoms. Even so, the large majority of extra-genital (and genital) infections in MSM and in women are asymptomatic and no solid evidence is present showing that symptoms are regularly associated with anorectal CT or pharyngeal CT or NG infections.

Hypothetically, anorectal infections in women could have an impact on the reproductive outcomes. This would be if an anorectal infection could be spread to the genital site. A recent study estimated that, when self-infection from the anorectal site plays a role, a less adequate treatment would sustain CT in a woman [76]. Thus anorectal infections would act as a reservoir (by self-infection, or during sexual activity) for genital infections (or re-infections). This would expand the spectrum of morbidity of anorectal infections in women, increasing the negative consequences of overlooking the anorectal infection. In genital CT, repeat infections have been linked to increased reproductive morbidity – more PID and more adverse reproductive outcomes; it is unknown if morbidity is also linked to repeat infection with extra-genital STI.

#### Avoided transmission

Pharyngeal and anorectal CT and NG are probably capable of being transmitted to genital sites of a partner [77, 78]. In women, infections may potentially be transmitted from their anorectal to genital site.

In the absence of better data on the transmission potential of extra-genital infections, any extra-genital STI that remains untreated should be considered transmissible. Given that there is a large number of extra-genital CT and NG currently missed by the current standard of care (i.e. untested, untreated or possibly sub-optimally treated), the transmission potential of such infections may be huge in women and in MSM. Infections at extra-genital sites may impact the total STI burden in the population by spread between sexual partners and even between a woman’s anatomic sites. A recent mathematical study in MSM suggest that oral sex has an important role in sustaining NG in MSM by providing a pool of untreated asymptomatic infection [79]. If extra-genital CT and NG infections in women and MSM do importantly help to sustain CT and NG endemicity, then the gaps in their current management could help explain why we do not see a decrease in CT and NG prevalence from surveillance data in the face of greatly increased genital testing and treating efforts [3, 4, 64].

Data on factors facilitating transmission are yet unavailable. It is for example unknown whether anorectal and pharyngeal bacterial load is associated with an increased risk for transmission, although such associations may be biologically plausible. The CT and NG load detected on pharyngeal or anorectal swabs load is generally lower than on genital swabs [32, 60, 75, 80–82]. Nevertheless, only few studies have yet directly compared bacterial loads between anatomic sites. It is unknown what are the transmission probabilities in relation to exposure, i.e. after exposure by an alternate anatomic site or after sex with an infected partner. A recent study among 51 women who reported anal sex and 90 MSM who reported anal sex found similar mean numbers and ranges in anorectal CT load [82]. This may suggest equal transmission probabilities in MSM and women reporting anal sex. Future mathematical models and prospective studies using multi-site sampling and measuring bacterial load, and culture to detect viable bacteria could help to obtain more insight in these aspects.

Missing extra-genital infections may also impact the transmission of other STI. In MSM, it has been shown that anorectal CT and NG facilitate HIV transmission [83–85]. Analyses even support the idea of a causal effect of incident anorectal STI on HIV diagnosis [86]. Given the high prevalences of anorectal CT and NG infections in MSM, it is not unreasonable to speculate that they may have played an important role in driving the HIV epidemic in these men. Therefore, anorectal CT and NG testing is a potentially cost-effective and scalable intervention to reduce HIV acquisition in high risk MSM [87, 88]. In populations with lower HIV prevalence, such as women in industrialized countries, such interventions may not be cost-effective. Yet, in women, an association between anorectal infections and HIV, whilst not yet studied, is plausible. This is because many studies have shown that the presence of genital STI is associated with increased HIV transmission in heterosexuals, and women have unprotected anal sex and anorectal STI, which are both acknowledged as important risk factors for HIV acquisition in MSM.

### Other extra-genital STI

As we have started to gain awareness of the occurrence of extra-genital CT and NG in women, we have been learning about other extra-genital STI as well. We know that *Mycoplasma genitalium* (MG), Herpes Simplex Virus, Human Papilloma Virus (HPV) and *Trichomonas vaginalis* (TV) can be found at the anorectal site in a woman. Presence at the anorectal site may be correlated to their presence in the vagina, such as has been shown for MG, TV and HPV [29, 89–91]. While some of these pathogens are routinely tested for at the genital sites of women, anorectal or pharyngeal testing of these STI is rarely done in practice. It is also not recommended and hampered when there are no available commercial assays (such as for MG). NAAT assays for the simultaneous detection of several anorectal infections ‘in one’ are being developed, and that perhaps may pose interesting future possibilities for combined extra-genital testing. The gaps that are revealed in the management of extra-genital CT and NG may also apply for these other STI. Further, detecting the simultaneous presence of different extra-genital pathogens will impact patient-management.

## Summary and conclusion

Extra-genital CT and NG, and perhaps other STI such as MG, may form a high potential for avoidable transmission and to some extent for avoidable morbidity in MSM and in women. Extra-genital CT and NG can be successfully detected using NAAT on simple to obtain self-taken samples. Both men and women are willing to test, and the so far hidden extra-genital STI epidemic can be revealed. Strategies can be taken to increase the reach of the testing and reduce costs by using internet based programs, e-health strategies and home-collection. Still several of the tests (NAAT testing on extra-genitally taken samples) and strategies (home-collection) are not FDA cleared. It is likely that, at least in the US, the needed paradigm shift from a genital-centered approach to testing (MSM and others at risk) to include a broader sampling frame, will not become routine until FDA clearance for extra-genital testing is obtained.

Testing should not solely be guided by indication of symptoms or behavioral exposure as the large majority of extra-genital STI are asymptomatic and over half of the extra-genital infections occur in the absence of (report of) such symptoms or behavioural exposures in both women and MSM. The majority of extra-genital infections in MSM are single site infections that are not being ‘inadvertently managed’ by treating urethral infections. Based on the current available data routine universal testing of all MSM instead of only testing MSM who report symptoms or behavioural exposure is therefore an imperative. To date, there is no evidence that the public health impact, in terms of transmission, of anorectal or pharyngeal infections is any different for women than it is for MSM. However, in women, the clinical implications of extra-genital infections, and gains by testing these, present uncertainties. In women, whether or not to implement routine testing would depend on the increment achieved in terms of avoided morbidity and further spread. For some less prevalent STI, such as NG in some countries, the increment is likely smaller in women than MSM due to the more infrequent occurrence of these infections in women. For more prevalent STI, such as CT and perhaps MG, the increment would depend on the number extra-genital infections that could be adequately managed (by treatment and partner management) above the number of infections that are already adequately managed by standard care for the genital infection. We lack essential information in several key area’s (summarized in Table 3) that need to be addressed to fully understand the scope of impact on public health and the clinical implications posed by the occurrence of extra-genital STI in women but also in MSM.

**Table 3:** Key research area’s in understanding the clinical and public health impact of extra-genital *Chlamydia trachomatis* (CT) and *Neisseria gonorrhoeae* (NG) infections in women and in men who have sex with men (MSM)

Key research area	Knowledge Gap on extra-genital CT and NG infections in women and MSM
Morbidity	Proctitis in anorectal infections
	Reproductive morbidity (women)
Efficacy control strategies: Treatment	Efficacy of treatment for both extra-genital and genital infections and associated factors (including treatment resistance, tissue absorption, duration of treatment to account for CT life cycle phases)
Efficacy control strategies: Re-testing, partner management	Efficacy of strategies to manage extra-genital infections (including strategies that are already taken to manage genital infections)
Transmission	Transmission risk between sexual partners and associated factors, such as bacterial load, sexual behavior
	Self-infection, i.e. transmission risk between anatomic sites within a patient and associated factors, i.e. bacterial load, sexual behavior (women)
	Role in the transmission of other STI including HIV
Detection	Microbiologic specifics of a CT - NAAT detection such as bacterial load, viability or other possible markers of transmission or of morbidity
Testing policy	Cost effectiveness of extra-genital CT and NG testing taking into account the key research area’s of efficacy of extra-genital control-strategies, transmission and morbidity

Future studies should address extra-genital and genital morbidity caused by extra-genital infections. Randomized controlled trials are needed to determine whether treatment efficacy for extra-genital infections differs from that of genital infections. The full spectrum including potential for further transmission of extra-genital infections to genital sites and related morbidity has not yet been evaluated in women. Prospective studies in couples or modeling studies could reveal more insight on what are the transmission probabilities between partners and between anatomic sites, and the associated factors. Morbidity and transmission may depend on the extent the bacteria is able to replicate or presents in high loads, which is unknown in MSM and in women. Studies using appropriate laboratory techniques, such as quantitative PCR for measuring bacterial load, and culture to detect viable bacteria and antibiotic resistance, to address these aspects are needed. These will greatly aid our understanding of extra-genital infections and the differences presented by these infections in MSM and women. Finally, cost-effectiveness of extra-genital testing has been evaluated for HIV infected MSM and was shown cost-effective in case of anorectal CT and NG [87, 88]. While it has been suggested that the lower prevalence of NG in women overall makes it more costly to screen for both CT and NG in women compared to MSM [15], this is in practice not likely a valid argument as most NAATs for CT are duplex assays also measuring NG with the same costs.

Hidden extra-genital infections might fuel the transmission of CT and NG and other STI and thereby increase the overall STI burden. This may be very important in the context of HIV and anorectal infection. Health care providers should be aware of the common occurrence of extra-genital infections. They should be doing more and urgently need a better understanding, especially in women, to obtain the highest gains of the increased CT and NG testing and treating efforts. Increased testing is an imperative for MSM. There is much to be learned to see if the same is true for women, and possibly for some heterosexual men as well.

### Ethics statement

Ethical approval was not required for this paper as it is a debate paper and not original research involving humans.

## Abbreviations

CDC: Centers for Disease Control and Prevention
CT: *Chlamydia trachomatis*
FDA: Food and Drug Administration
HPV: Human Papilloma Virus
NAAT: Nucleic Acid Amplification Test
NG: *Neisseria gonorrhoeae*
MG: *Mycoplasma genitalium*
MSM: Men who have sex with men
TV: *Trichomonas vaginalis*
